# Multiparametric-MRI habitat radiomics analysis for discriminating pathological types of brain metastases

**DOI:** 10.3389/fonc.2025.1714315

**Published:** 2025-12-02

**Authors:** Jinling Zhu, Xin Xie, Jixuan Deng, Ruizhe Xu, Li Zou, Ye Tian, Wu Cai, Bo Zhang

**Affiliations:** 1 Department of Radiology, The Second Affiliated Hospital of Soochow University, Suzhou, China; 2 Department of Radiotherapy & Oncology, The Second Affiliated Hospital of Soochow University, Suzhou, China

**Keywords:** magnetic resonance imaging, habitat radiomics, brain metastases, lung cancer, breast cancer, gastrointestinal cancer

## Abstract

**Background:**

Early identification of the primary tumor types in brain metastases (BMs) is crucial for developing effective treatment strategies. This study aimed to evaluate the potential of multiparametric MRI (mpMRI)-based habitat radiomics analysis in differentiating the pathological types of BMs.

**Materials and methods:**

Pre-treatment MR images from 328 BMs patients at a single center were retrospectively collected and randomly divided into a training set (229 cases) and a test set (99 cases). Tumor regions were manually segmented on contrast-enhanced T1-weighted images (CE-T1WI), and the K-means clustering algorithm was employed to classify the tumor into four distinct sub-regions. Radiomics features were extracted separately from each sub-region to construct the habitat model. The resulting habitat model was compared alongside a traditional whole-tumor radiomics model, a clinical model, and a combined model (integrating habitat and clinical variables). Model performance was evaluated by the area under the receiver operating characteristic curve (AUC), as well as accuracy.

**Results:**

The combined model achieved the highest overall performance (training AUC: 0.992, accuracy: 0.952; test AUC: 0.939, accuracy: 0.845), outperforming the habitat model (training AUC: 0.965, accuracy: 0.876; test AUC: 0.888, accuracy: 0.835), traditional radiomics model (training AUC: 0.984, accuracy: 0.866; test AUC: 0.884, accuracy: 0.754), and clinical model (training AUC: 0.788, accuracy: 0.731; test AUC: 0.716, accuracy: 0.653). However, class-specific evaluation revealed substantial performance variation, with F1-scores of 0.874 for lung cancer BMs, but only 0.333 and 0.200 for breast and gastrointestinal cancer BMs, respectively.

**Conclusions:**

This study demonstrates that while habitat radiomics shows potential for classifying BMs, its current performance is constrained by class imbalance and scanner heterogeneity. Consequently, our primary contribution lies in providing a critical baseline and a clear direction, prioritizing data-centric solutions as the essential next step for the field.

## 1 Introduction

Brain metastases (BMs) represent the most common malignant tumors of the central nervous system in adults, with an incidence at least tenfold greater than that of primary brain tumors (1, 2). These lesions significantly impair patient prognosis and quality of life and are major causes of morbidity and mortality among cancer patients. The estimated incidence of BMs ranges from 20% to 40% (3). Among these cases, the majority originate from lung cancer (LC) (41–56%), breast cancer (BC) (13–30%), malignant melanoma (MM) (6–11%), or gastrointestinal cancer (GIC) (6–9%) (4). However, in up to 15% of BMs patients, the primary tumor remains unidentified; these cases are classified as cancers of unknown primary (CUP) type (5, 6). Despite the widespread application of systemic diagnostic methods to detect primary tumors, CUP cases persist (6).

While tissue sampling remains the cornerstone of brain tumor diagnosis, the contemporary gold standard has evolved to an integrated approach that synthesizes histopathological examination with molecular genetic profiling, as reflected in the World Health Organization classification (7). However, this invasive procedure carries inherent limitations, such as intracranial hemorrhage and patient intolerance, while the process of obtaining and analyzing tissue can be time-consuming, potentially delaying critical therapeutic interventions. Therefore, the development of a rapid and noninvasive differential diagnostic method for BMs is crucial to optimize clinical decision-making and improve patient outcomes. Magnetic resonance imaging (MRI) is the standard neuroimaging modality for evaluating and diagnosing BMs; however, accurate identification of the pathological type of BMs remains a significant challenge because of the lack of specific imaging biomarkers.

Previous studies have demonstrated that radiomics models can effectively predict the primary origin of BMs, assess gene mutation status, and evaluate therapeutic responses (8–11). Although whole-tumor radiomics analyses provide partial quantification of intratumoral heterogeneity, they often assume a homogeneous tumor structure. In reality, variations in perfusion and other physiological factors within the tumor can give rise to distinct spatial subregions with unique structural and biological characteristics, referred to as "habitats" (12, 13). To address this spatial heterogeneity, habitat imaging technology has emerged as a novel approach. Unlike traditional radiomics models, habitat-based analysis focuses on extracting features from specific tumor subregions. By clustering voxels with similar imaging and biological properties, this method enables more precise quantification of regions closely associated with tumor growth and invasiveness, thereby enhancing the characterization of intratumoral heterogeneity (14, 15). In recent years, habitat radiomics analysis has become a promising research direction and has been initially applied to the prognostic assessment of fields such as breast cancer (16), glioma (17), colorectal cancer (18), lung cancer (19), and nasopharyngeal carcinoma (20).

Recent studies have highlighted the potential of habitat radiomics analysis in predicting the prognosis of BMs patients (21). However, the application of multiparametric MRI (mpMRI)-based habitat radiomics models in the pathological classification of BMs has not been fully explored. The imaging phenotypes analyzed in this study reflect the macroscopic structure of established BMs, not the cellular processes of local invasion. Our focus is on the radiomics features of these lesions, which result from the successful colonization and proliferation of metastatic cells in the brain microenvironment, forming distinct habitats with varying physiological properties. To comprehensively evaluate the predictive performance of this habitat model, we compared its predictive performance with that of the conventional whole-tumor radiomics model and clinical model.

## 2 Materials and methods

### 2.1 Study population

**Figure 1:**
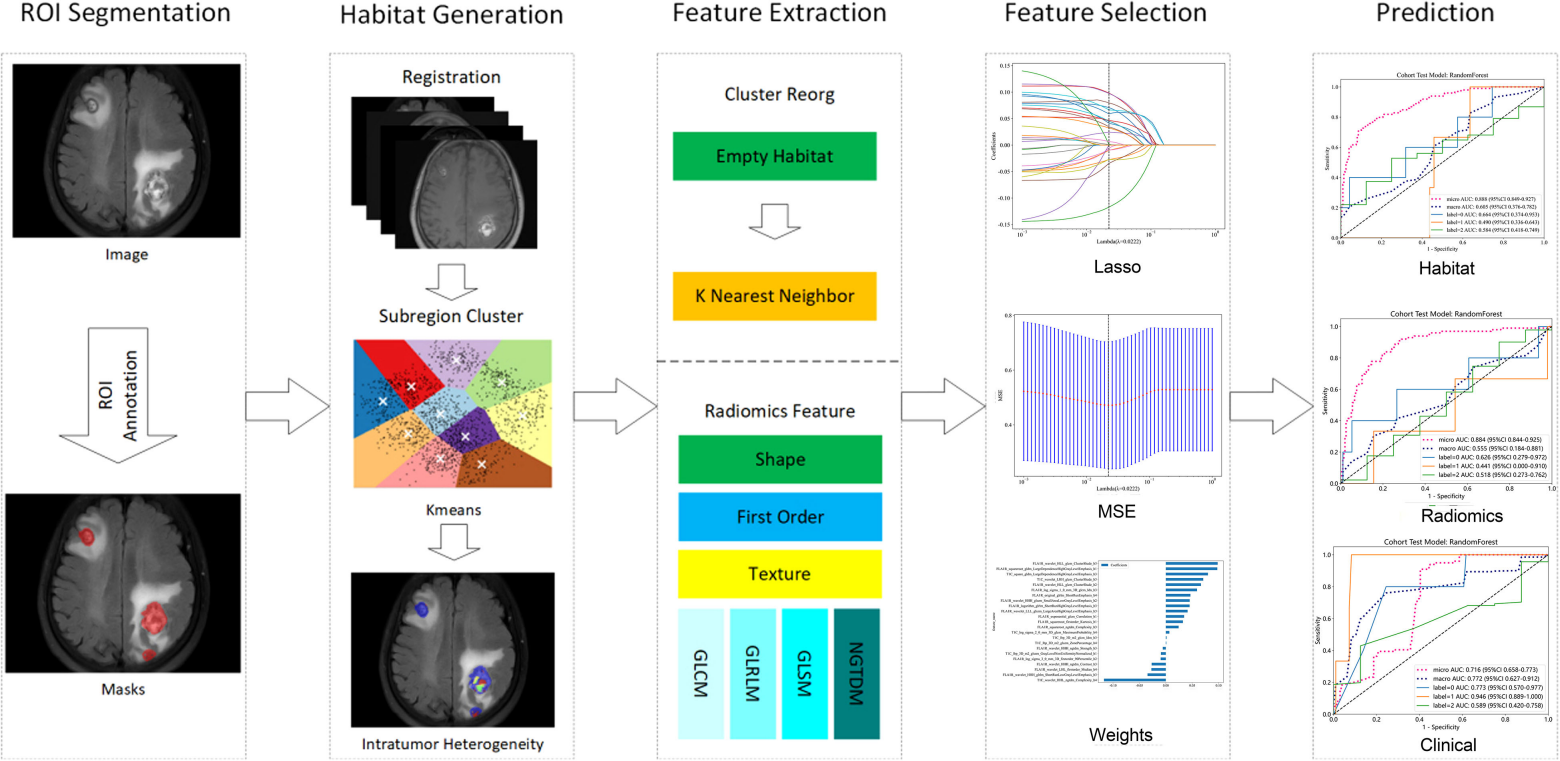
Workflow of habitat radiomics analysis.

This retrospective study was approved by the Institutional Ethics Committee of the hospital, and the requirement for informed consent was waived. The study design and workflow are shown in **Figure 1**. The clinical data and MR images of BMs patients admitted to our hospital from December 2010 to April 2023 were collected, and the inclusion and exclusion criteria were determined. The inclusion criteria were as follows: (1) BMs confirmed by histopathology or imaging examination and clinical follow-up. (2) BMs patients who did not receive treatment (radiotherapy, surgery, chemotherapy or targeted therapy). (3) The primary tumor was confirmed by histopathology. (4) Complete clinical and imaging data of the patients. The exclusion criteria were as follows: (1) Combined with other malignant tumors. (2) The maximum lesion diameter of less than 5 mm. (3) Poor image quality. The outcome predicted by the model was the pathological types of BMs, which was classified into one of three categories: lung cancer, breast cancer, or gastrointestinal cancer. The definitive diagnosis for each patient was determined through histopathological analysis of tissue obtained *via* neurosurgical resection or biopsy. In cases where tissue from the BMs was unavailable, the diagnosis was confirmed *via* immunohistochemical profiling of the primary tumor and further supported by clinical and imaging evidence specific to that primary cancer.

The collected patient characteristics included clinical variables (age, sex) and imaging features (number of tumors, maximum tumor diameter, and maximum diameter of the peritumoral edema zone).

### 2.2 Imaging acquisition protocol

The MRI dataset consists of four different imaging sequences: T1-weighted image (T1WI), Contrast-enhanced T1-weighted image (CE-T1WI), T2-weighted image (T2WI), and T2-weighted fluid attenuated inversion recovery (FLAIR). The contrast agent used in the CE-T1WI scan was gadopentetate dimeglumine, which was administered intravenously at a dose of 0.1 mmol/kg. The scanning parameters of the MR images can be found in **Supplementary Material S1**.

### 2.3 Image preprocessing and tumor segmentation

Before manually segmenting the region of interest (ROI), the raw image was preprocessed as follows: (1) To standardize the spatial resolution of all imaging datasets, the voxel spacing was resampled and adjusted to 1 mm×1 mm×1 mm. (2) The N4 bias field correction algorithm was used to normalize the intensity by eliminating low-frequency intensity changes (22). (3) Rigid registration of CE-T1WI images with T2WI, T1WI and FLAIR images was performed to ensure spatial correspondence between different sequence images.

Finally, the entire tumor ROI was identified layer-by-layer on CE-T1WI and manually delineated by a junior radiologist *via* the open-source software ITK-SNAP (version 3.8.0). To minimize interobserver bias, these delineations were subsequently evaluated by a second radiologist with over 15 years of experience. To avoid interobserver bias in the segmentation process, the two radiologists who delineated the tumor ROI and habitats were blinded to the final pathological diagnosis of the BMs. All the images were presented in a random order and labeled only with a unique study identifier that contained no diagnostic information.

### 2.4 Subregion generation

**Figure 2:**
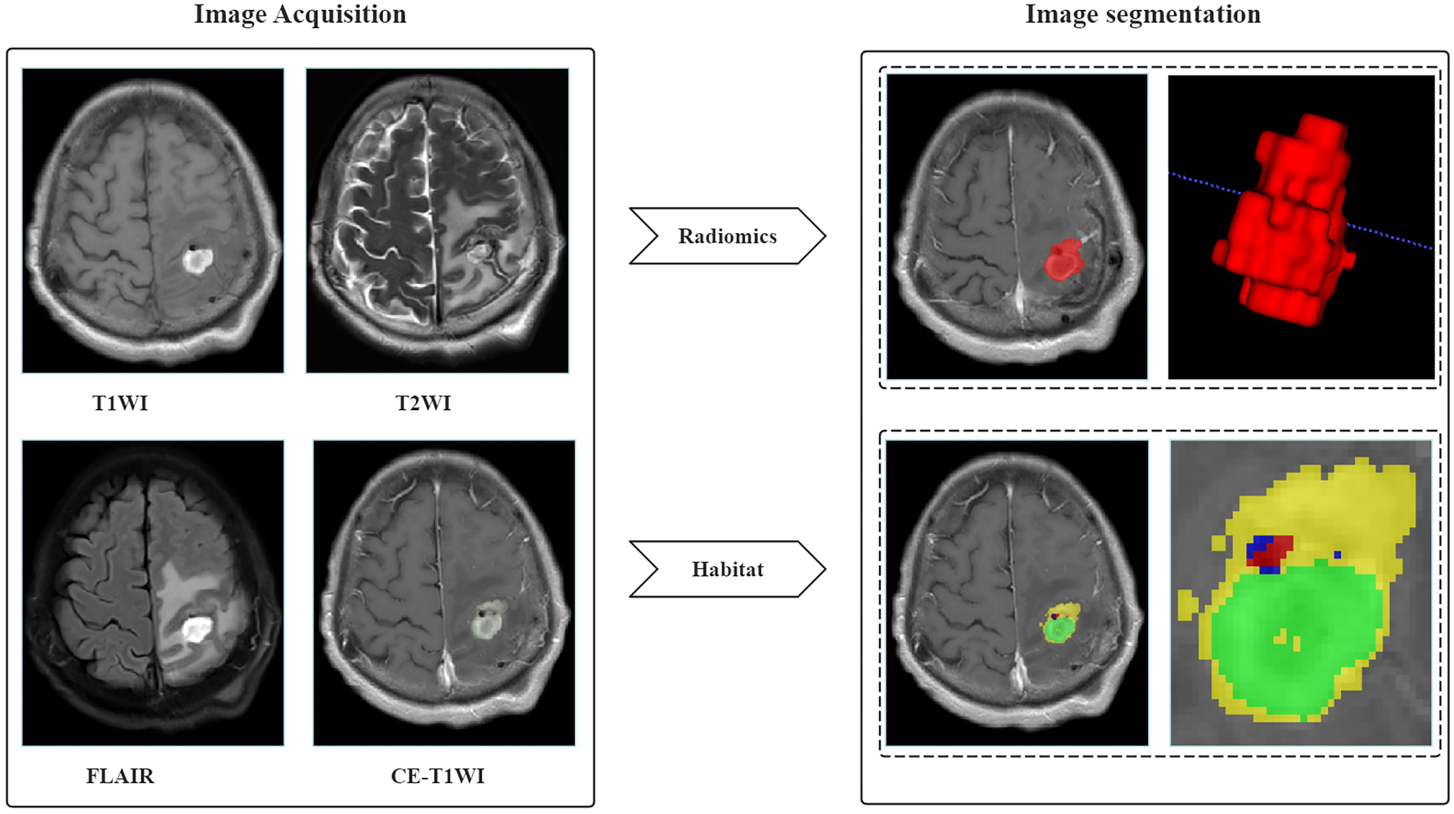
Segmentation methods of the habitat model and radiomics model.

This study employed the K-means method for subregional clustering, using the squared euclidean distance between voxel intensities as the similarity metric. All voxels were assigned to specific clusters and visualized as spatial habitats (23). We explored cluster numbers ranging from 2-10, with the optimal number determined by the Calinski–Harabasz (CH) score (24). Notably, the habitat model and radiomics model utilized distinct segmentation approaches, as illustrated in **Figure 2**.

### 2.5 Radiomics feature extraction and screening

Features were extracted separately from tumor subregions and the original whole tumor *via* PyRadiomics, with four radiomics feature categories obtained from each sequence: (1) shape/size-based features; (2) first-order features; (3) textural features; and (4) wavelet-based features. Since subregional features were derived from voxel-value clustering, shape- and size-based features were excluded. The extraction of radiomics features was performed *via* an automated script. However, the parameter tuning for the habitat definition (e.g., the choice of clustering algorithm parameters) was conducted by an engineer who was blinded to the clinical outcomes to prevent data snooping and overfitting. For each subregion, corresponding radiomics features were extracted and labeled sequentially (e.g., "feature_h1"). Regions with insufficient voxel counts were processed *via* a k-nearest neighbor approach to maintain data completeness across imaging modalities.

### 2.6 Feature selection

After feature extraction, feature screening and dimension reduction were carried out as follows: (1) The Z score method was used to standardize all the features. (2) The intraclass correlation coefficient (ICC) was used to evaluate the repeatability and stability of the radiomics features. (3) The T-test was used to retain the features with P values less than 0.05 for subsequent analysis. (4) Spearman’s rank correlation coefficient was calculated between the features. If the correlation coefficient exceeded 0.9 between any two features, only the more discriminative feature was retained. (5) The least absolute shrinkage and selection operator (LASSO) was applied to identify the optimal feature subset (25). The optimal regularization parameter (λ) was determined *via* 10-fold cross-validation, and features with nonzero coefficients were selected for model construction.

### 2.7 Model construction and validation

To improve model reliability and reduce sample bias, repeated randomization was conducted until no significant differences were observed in any of the features between the training and test sets (P > 0.05). During model training, the class_weight parameter (26) was adjusted on the basis of the label distribution: the weights for GIC and BC were increased fivefold while maintaining the original weight of the LC to mitigate class imbalance effects. To ensure model robustness, 5-fold cross-validation was implemented during training, and hyperparameters were optimized through a grid search. The random forest (RF) algorithm demonstrates good adaptability to imbalanced datasets and multiclass classification tasks, as its ensemble voting mechanism effectively balances class predictions while improving model robustness and reliability (27). Accordingly, four classification models were constructed using the selected features with the RF algorithm (1): a clinical model incorporating clinical parameters (age, sex, number of tumors, maximum tumor diameter, and maximum edema diameter); (2) a traditional radiomics model based on whole-tumor features; (3) a habitat model utilizing habitat subregional radiomics features; and (4) a combined model integrating both habitat and clinical features.

The discriminative performance of the multiclass model was assessed *via* an independent test cohort. No substantial differences were observed between the development and validation cohorts with respect to the data source, eligibility criteria, outcome assessment, or imaging protocols, as both cohorts were derived from the same patient population at the same institution. All procedures for image preprocessing, ROI delineation, feature extraction, and model application were consistent with those employed during the development phase. The predictions for the test cohort were obtained by applying the finalized model to the processed test data without any adjustments. For each case, the model generated a vector consisting of three probability estimates, indicating the predicted likelihood of the BMs originating from lung cancer, breast cancer, or gastrointestinal cancer. The sum of these three probabilities for each case equals 1. The final predicted class label was determined on the basis of the maximum probability rule. Specifically, the class (LC, BC, or GIC) associated with the highest predicted probability was selected as the model's prediction.

### 2.8 Statistical analysis

In this study, the Shapiro–Wilk test was used to assess the normality of the clinical characteristics. For quantitative variables, the T-test (for normally distributed data) or the Mann–Whitney U test (for nonnormally distributed data) was used for analysis. For qualitative variables, the chi-square test or Fisher's test was used to evaluate their significance. A two-sided P < 0.05 indicated a statistically significant difference. The classification performance of the model was evaluated by calculating the micro average area under the curve (Micro-AUC) and its 95% confidence interval (95% CI), accuracy (ACC), sensitivity (SEN), specificity (SPE), positive predictive value (PPV), and negative predictive value (NPV). Data analysis and the development of machine learning models were conducted *via* Python (version 3.7.12), Onekey (version 3.3.5), and scikit-learn (version 1.0.2).

## 3 Results

### 3.1 Clinical parameters

A total of 1514 patients were initially screened. On the basis of the inclusion and exclusion criteria, 328 patients with BMs, comprising a total of 1357 metastatic lesions, were ultimately enrolled in this study. Among them, there were 259 LC patients with 1098 metastatic lesions, 32 BC patients with 153 metastatic lesions, and 37 GIC patients with 106 metastatic lesions. All the samples were randomly divided into a training set (N = 229) and a test set (N = 99) at a ratio of 7:3. **Table 1** presents the clinical characteristics of the patients in both groups. This study ultimately enrolled 328 patients, comprising 208 males and 120 females, with ages ranging from 32-85 years (mean age: 63.17 ± 10.54 years). Univariate intergroup comparison revealed no statistically significant differences (P > 0.05) between the two groups in terms of age, sex, number of tumors, maximum tumor diameter, or maximum edema diameter.

**Table 1:** Patients’ clinical characteristics in the training and test cohort.

Features name	All	Training cohort	Test cohort	P value
Age	63.17 ± 10.54	62.88 ± 10.07	63.85 ± 11.57	0.247
Tumor maximum diameter (mm)	23.25 ± 11.64	22.92 ± 12.14	24.02 ± 10.40	0.145
Number of tumors	4.14 ± 5.29	4.05 ± 5.48	4.33 ± 4.83	0.296
Maximum diameter of peritumoral edema (mm)	40.95 ± 28.29	39.02 ± 27.53	45.43 ± 29.63	0.077
Sex				0.353
Male	208(63.41%)	141(61.57%)	67(67.68%)	
Female	120(36.59%)	88(38.43%)	32(32.32%)	

### 3.2 Subregion cluster

**Figure 3:**
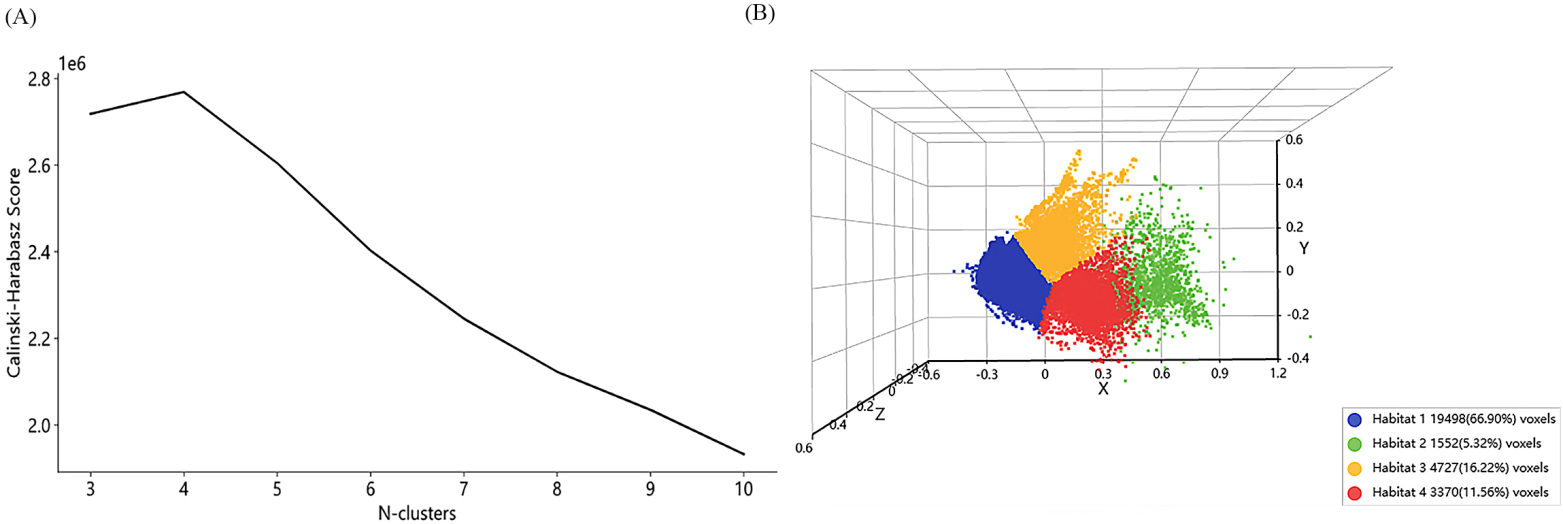
Calinski–Harabasz score for different clusters **(A)** and visualization characteristics for 4 clusters **(B)**.

The CH index was calculated for each variable with K values between 2 and 10, and the optimal number of clusters was determined by plotting the contours of the cluster analysis. As the number of clusters increases, the optimal number of clusters K=4 corresponds to the turning point of the curve. Each sample was divided into four different subregions (Habitat 1, Habitat 2, Habitat 3, and Habitat 4) *via* K-means clustering. The scores of the different clusters and the features of the four clusters are visualized in **Figure 3**.

### 3.3 Screening of subregion and traditional radiomics features

**Figure 4:**
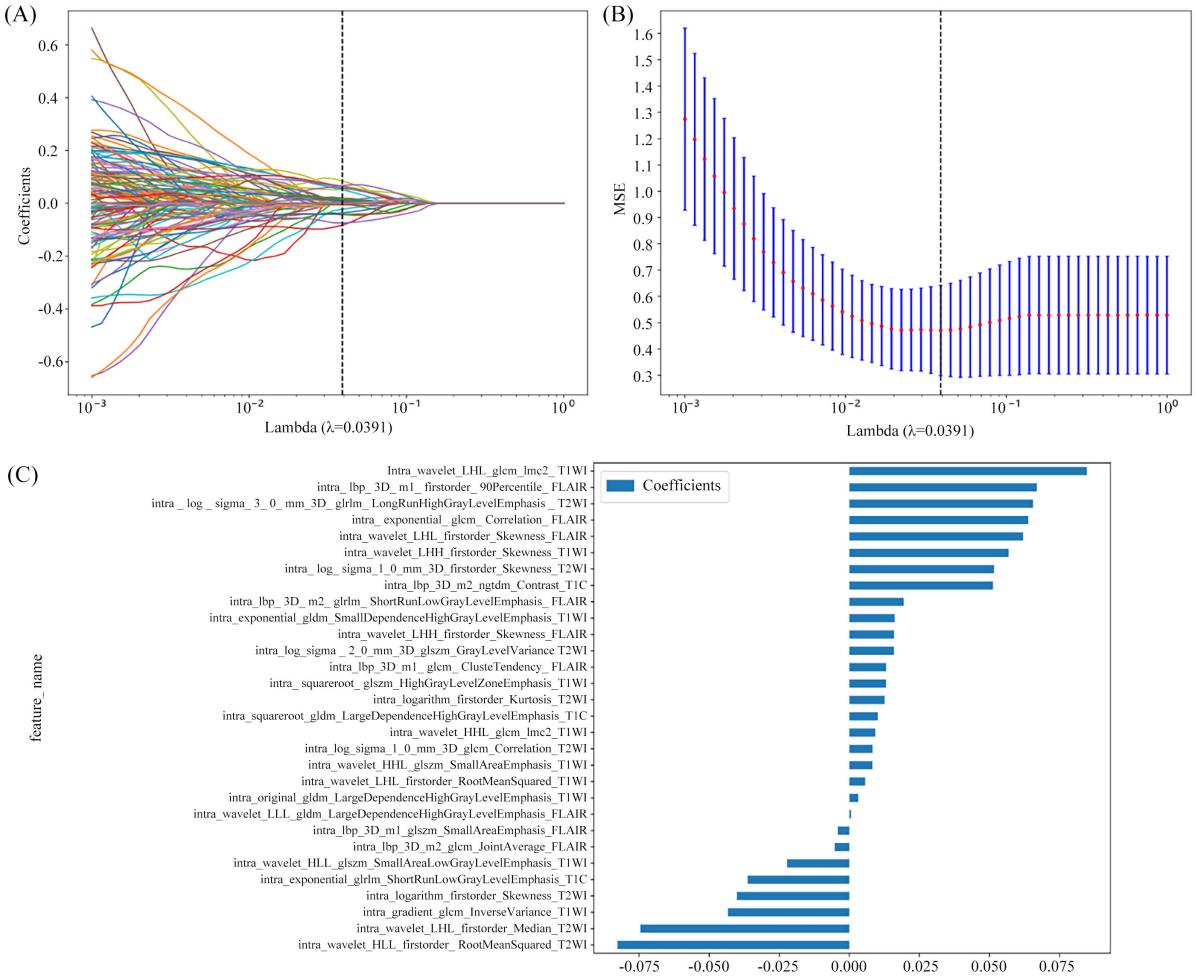
Radiomics feature selection based on the LASSO algorithm **(A)**. Tenfold cross-validation coefficients and MSE **(B)**. Histogram based on the selected features **(C)**.

For the radiomics model, a total of 7,336 radiomics features were extracted from the whole tumor volumes across four image sequences. Following feature selection *via* LASSO regression, 30 features with the highest predictive value were retained for model construction. These comprised 8 features from T2WI, 10 features from T1WI, 3 features from CE-T1WI, and 9 features from FLAIR sequences. The feature selection process is illustrated in **Figure 4**.

**Figure 5:**
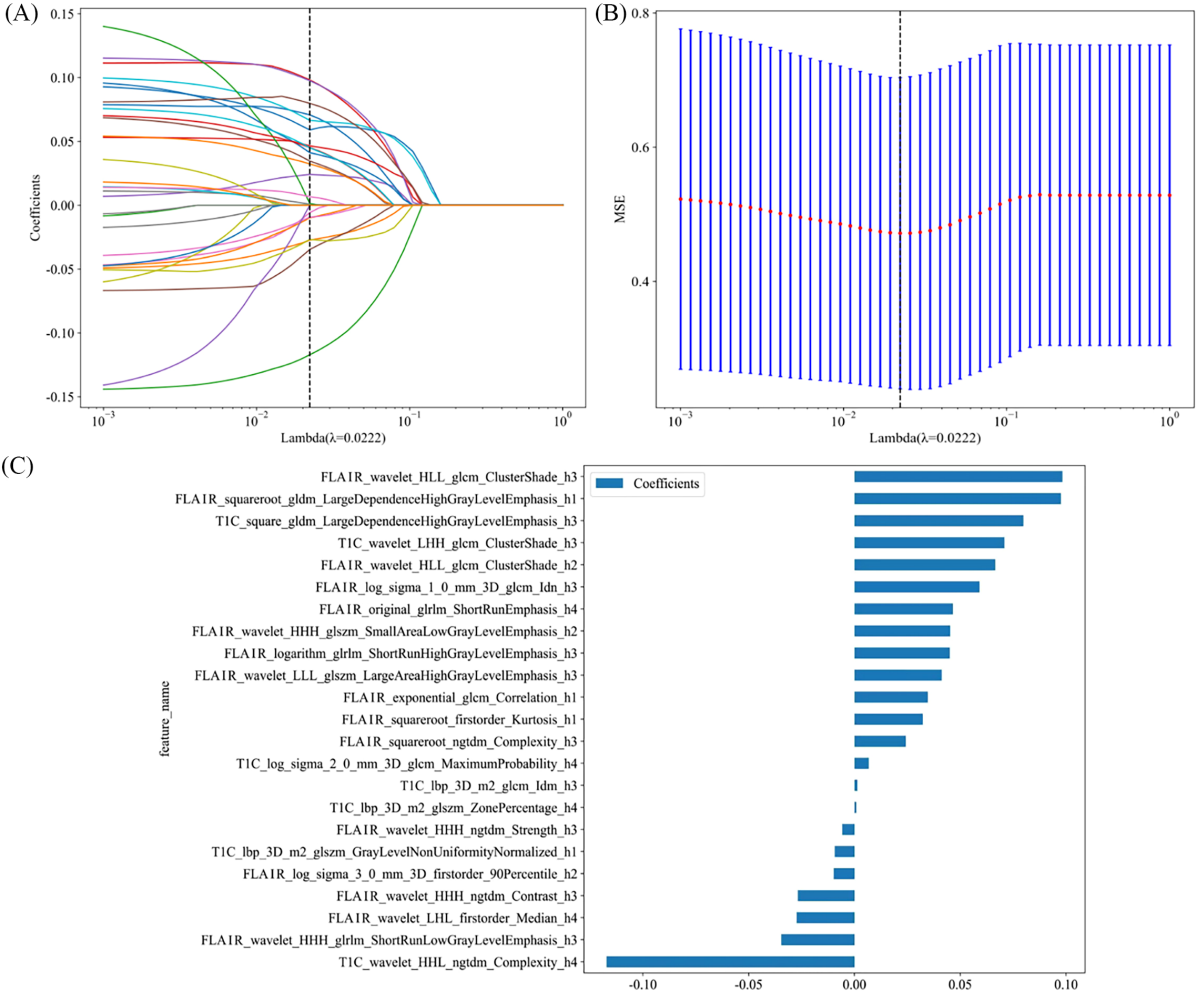
Habitat feature selection based on the LASSO algorithm **(A)**. Tenfold cross-validation coefficients and MSE **(B)**. Histogram based on the selected features **(C)**.

For the habitat model, a total of 29,344 radiomics features were extracted from different subregions across the four image sequences. Following feature selection *via* LASSO regression, 23 subregion-based habitat features contributing most significantly to the habitat model were identified. Among these, 16 features were derived from the FLAIR sequence, and 7 features were derived from the CE-T1WI sequence. The feature selection process for the habitat features is illustrated in **Figure 5**.

### 3.4 Prediction model development and validation

#### 3.4.1 Clinical model

The model developed using clinical features (age, sex, number of tumors, maximum tumor diameter, and maximum diameter of edema) achieved an AUC of 0.788 (95% CI: 0.755–0.821) and an accuracy of 0.731 in the training cohort. In the test cohort, it demonstrated an AUC of 0.716 (95% CI: 0.658–0.773), with an accuracy of 0.653.

#### 3.4.2 Traditional radiomics model

This model achieved an AUC of 0.984 (95% CI: 0.977–0.991) and an accuracy of 0.866 in the training cohort. Its performance decreased in the test cohort, yielding an AUC of 0.884 (95% CI: 0.844–0.925) and an accuracy of 0.754.

#### 3.4.3 Habitat model

In the training cohort, the habitat model demonstrated a high AUC value of 0.965 (95% CI: 0.953–0.976) and an accuracy of 0.876. It exhibited relatively stable performance in the test cohort, achieving an AUC of 0.888 (95% CI: 0.849–0.927) and an accuracy of 0.835.

#### 3.4.4 Combined model

The integrated model incorporating both clinical and radiomics features yielded an AUC of 0.992 (95% CI: 0.988–0.996) and an accuracy of 0.952 in the training cohort. In the test cohort, the model achieved an AUC of 0.939 (95% CI: 0.914–0.964) and an accuracy of 0.845.

#### 3.4.5 Model comparison

**Figure 6:**
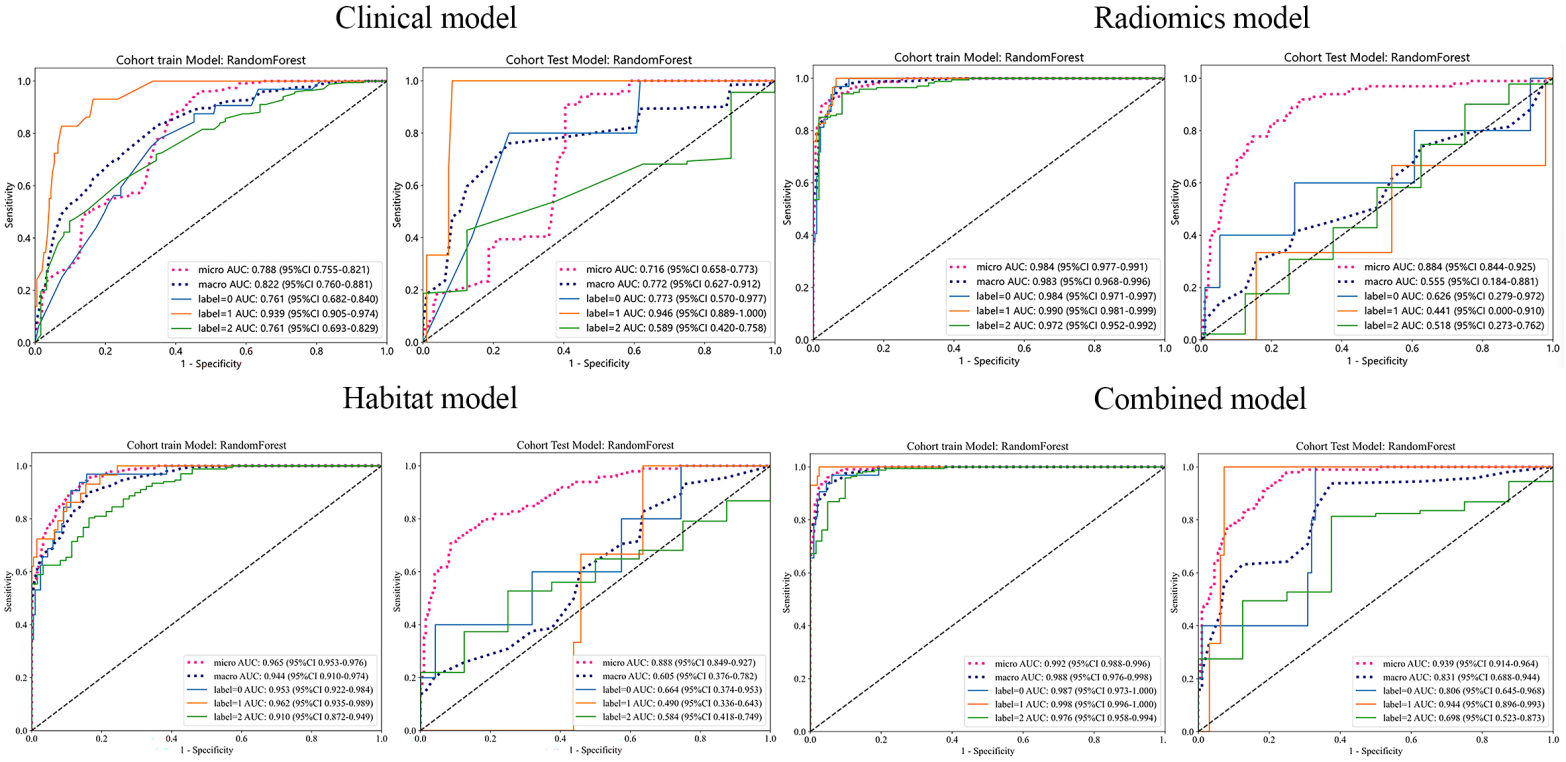
ROC curves of different models in the training and test cohorts. Label 0, gastrointestinal cancer; label 1, breast cancer; label 2, lung cancer.

As illustrated in **Figure 6**, **Table 2**, the combined model demonstrated the best overall performance metrics (AUC and accuracy) in both the training and test cohorts, outperforming any of the individual models alone. The precision, recall, F1 score, and other metrics for each category can be found in the **Supplementary Material S2**.

**Table 2:** A comparative analysis of the performance of various models.

Model	ACC	AUC	95% CI	SEN	SPE	PPV	NPV	Recall	Cohort
Clinical	0.731	0.788	0.755-0.821	0.493	0.849	0.621	0.770	0.493	Train
	0.653	0.716	0.658-0.773	0.374	0.793	0.474	0.717	0.373	Test
Radiomics	0.866	0.984	0.977-0.991	0.611	0.993	0.979	0.836	0.611	Train
	0.754	0.884	0.844-0.925	0.313	0.975	0.861	0.739	0.313	Test
Habitat	0.876	0.965	0.953-0.976	0.952	0.838	0.747	0.972	0.952	Train
	0.835	0.888	0.849-0.927	0.727	0.889	0.766	0.867	0.727	Test
Combined	0.952	0.992	0.988-0.996	0.965	0.945	0.898	0.982	0.965	Train
	0.845	0.939	0.914-0.964	0.909	0.813	0.709	0.947	0.909	Test

## 4 Discussion

To more accurately quantify intratumoral heterogeneity, we conducted a habitat radiomics analysis by segmenting tumors into subregions on the basis of clusters of similar voxel characteristics. In this study, BMs were segmented into four distinct subregions *via* the K-means clustering algorithm, and radiomics features extracted from each subregion were utilized to construct a habitat-based radiomics model. Concurrently, whole-tumor radiomics models, clinical models, and combined models were developed for discriminating among the pathological types of BMs (LC, BC, and GIC). The results indicated that the habitat model demonstrated superior accuracy compared with the traditional radiomics model and the clinical model. We observed a slight degradation in the performance of the habitat model on the test set (training set AUC: 0.965 vs. test set AUC: 0.888), which is an expected phenomenon in predictive modeling as the model encounters previously unseen variations in new data. Notably, the combined model exhibited the highest predictive performance for differentiating the pathological types of BMs, with an AUC of 0.939 and accuracy of 0.845 on the test set. This highlights the importance of integrating imaging features with clinical data.

The present study demonstrates that habitat radiomics analysis, by quantifying spatial heterogeneity within BMs, enhances the non-invasive classification of their pathological types. Our approach diverges from conventional whole-tumor or peritumoral region-based analyses by focusing on distinct intratumoral subregions. This methodology enabled the development of a model that achieved an AUC of 0.939 on an independent test set. Such performance gain can be attributed to the model's capacity to capture heterogeneous biological information that is often averaged out in whole-tumor analyses. Furthermore, the habitat radiomics analysis has been shown to effectively correlate with key biological behaviors, such as tumor growth patterns and invasiveness, which are essential for understanding tumor biology (15, 28). Our findings are contextualized by prior efforts in BMs radiomics. While previous studies utilizing whole-tumor or peritumoral features have reported AUC ranging from 0.64 to 0.87 (8, 29, 30), their approach may overlook critical intratumoral heterogeneity. The performance of our habitat-based model, developed with a cohort of 328 patients—a sample size situated within the mid-to-upper range of comparable studies (8, 9, 29, 31–33)—indicates that the habitat radiomics analysis holds promise for providing a more granular analysis of tumor subregions. On the independent test set, the model achieved an overall Micro-AUC of 0.939 and a corresponding Micro-F1 score of 0.796. Nevertheless, a class-specific analysis uncovered a key limitation driven by a deliberate trade-off: to address the class imbalance, the class_weight adjustment prioritized sensitivity for minority classes, resulting in high recall but low precision and consequently low F1-scores (0.333 for BC and 0.200 for GIC) (34). The model's failure on minority classes, combined with scanner heterogeneity, constitutes a major challenge. This suggests that future studies cannot simply apply the existing pipeline but must prioritize addressing data imbalance and heterogeneity. Within this context, our study provides a dual contribution: First, it offers a proof of concept that a more precise quantification of spatial heterogeneity can enhance diagnostic performance, validating the promise of habitat radiomics analysis. Second, and perhaps more importantly, it delivers a critical warning and a clear path forward: the full potential of this habitat-based radiomics will only be realized through a paradigm shift that prioritizes solving data-centric challenges—imbalance and heterogeneity—alongside model development.

Recent studies have demonstrated that habitat radiomics analysis provides meaningful insights into tumor aggressiveness and treatment response. Lee et al. (21) conducted a retrospective analysis of MRI data from 52 patients with BMs, identifying three structural and three physiological MRI-derived habitats. Subregions characterized by low vascularity and low solid enhancement were found to be indicative of viable tumor tissue, suggesting their potential as therapeutic targets. A follow-up study (35) linked low vascularity and low solid enhancement subregions to posttreatment recurrence risk. In the present study, structural segmentation of the BMs was performed *via* conventional MRI sequences. We propose the following hypothetical biological interpretations for each identified subregion: Habitat 1 is hypothesized to predominantly represents an ischemic necrotic or cystic core. The voxel-level features extracted from this region demonstrated the highest contribution weights. This finding is consistent with the well-established pathological feature of BMs, which has a high propensity for cystic degeneration and necrosis. Habitat 2 may reflect a hemorrhagic component, as suggested by its relatively high signal intensity across multiple MRI sequences and lack of significant enhancement on CE-TIWI sequence. Habitat 3 is postulated to correspond to the solid tumor component, potentially associated with areas of active tumor cell proliferation. The characteristics of this habitat are likely to reflect the tumor's core biological behavior. Feature importance analysis revealed that attributes derived from this habitat made the most substantial contribution to the predictive performance of the model, highlighting their critical role in assessing tumor biology. Habitat 4 appears to represent solid tissue exhibiting T2WI sequence hypointensity and weak enhancement. These imaging features suggest a tumor subpopulation characterized by low angiogenic activity and a predominantly infiltrative growth pattern, which may link to therapy resistance and recurrence (35). It is important to note that pathological or pathophysiological validation of these habitat assignments was not feasible within the scope of the current study. Future investigations incorporating advanced functional imaging techniques and pathomics data are necessary to further validate these findings and substantiate the proposed biological interpretations.

Habitat radiomics analysis has been primarily applied in oncology to assess treatment response following radiotherapy. More recently, this approach has shown promise in tumor classification. For example, Shen et al. (36) used habitat radiomics to subtype lung cancer histopathologically, achieving an AUC of 0.916, which underscores its potential for discriminating tumor subtypes. In contrast, conventional radiomics analysis typically encompasses the entire tumor volume, including necrotic or predominantly benign regions. These areas may obscure the true heterogeneity within the tumor, thereby limiting diagnostic accuracy. Although the habitat model in our study demonstrated only a slight improvement in the AUC compared with conventional radiomics in the test set (0.888 vs. 0.884), it exhibited meaningful gains in accuracy (0.835 vs. 0.754) and sensitivity (0.727 vs. 0.313). These findings are consistent with those of previous studies (14, 37, 38) that support the general superiority of habitat radiomics in capturing tumor heterogeneity. Our model captures heterogeneous radiomics signatures from MR data and shows potential for pathological subtyping of BMs in specific categories. Clinically, this model could be implemented as a decision-support tool in the radiology workflow. When a BM is detected, the model could provide a probabilistic classification of the primary origin, thereby guide subsequent targeted diagnostic tests and accelerate the initiation of site-specific therapies (e.g., bronchoscopy for suspected lung cancer, mammography for suspected breast cancer), especially in cases where biopsy is high-risk or unfeasible. Such a tool may accelerate the initiation of site-specific therapy and improve diagnostic efficiency.

Furthermore, the most discriminative features contributing to the habitat model were derived predominantly from the CE-T1WI and FLAIR sequences. This observation likely reflects the complementary advantages of these sequences in capturing tumor microenvironment heterogeneity: while conventional T1WI and T2WI provide detailed anatomical information, their spatial features are largely redundant with those of CE-T1WI and FLAIR. CE-T1WI offers superior delineation of tumor enhancement patterns, whereas FLAIR more accurately delineates peritumoral signal abnormalities. This finding is consistent with prior research. Charron et al. (39) demonstrated that the combination of CE-T1WI and FLAIR improves the accuracy of BMs detection. Similarly, Zhao et al. (40) established effective models for distinguishing brain tumor subtypes *via* these sequences. Collectively, these findings reinforce the foundational role of CE-T1WI and FLAIR in radiomics research on brain tumors.

This study has several limitations: (1) The single-center retrospective design may limit the external validity of our findings, as patient demographics and imaging protocols at one institution may not be representative of other clinical settings. Consequently, the generalizability of the radiomics model requires further validation across multiple centers and diverse populations. (2) Despite preprocessing and feature stability screening, potential bias from multi-scanner and acquisition parameter heterogeneity may persist, warranting future application of advanced harmonization techniques. (3) Manual delineation of ROI is time-consuming and laborious, and an automatic segmentation method needs to be developed. (4) There is a deficiency in the validation of image–pathology spatial colocalization. (5) This study did not encompass patients with melanoma BMs. The primary reason is that the annual incidence of melanoma in Asia is extremely low, at less than 0.001% (41). Furthermore, our study is limited by a notable class imbalance among the included cancer types. The uneven distribution of cases, which reflects the real-world epidemiology of BMs, directly contributed to the model's suboptimal performance, particularly the low F1-scores observed for the minority classes (breast and gastrointestinal cancers).

Our future research will focus on a prospective, multicenter cohort study designed to collect data from diverse populations and imaging protocols, with an emphasis on achieving a more balanced representation across tumor types. The refined model will subsequently undergo rigorous external validation in this independent cohort to comprehensively evaluate its generalizability and critically assess its clinical utility in real-world settings.

## 5 Conclusion

In conclusion, while our habitat radiomics analysis shows potential for identifying brain metastases from lung cancer, it demonstrated significant limitations in classifying the minority classes (breast and gastrointestinal cancers), primarily due to substantial class imbalance and scanner heterogeneity. Therefore, the principal contribution of this work is not a fully validated diagnostic tool, but rather a critical baseline and cautionary tale. It underscores that resolving data-centric challenges is an essential prerequisite before such models can be reliably applied in heterogeneous clinical settings.

## Data Availability

The data analyzed in this study is subject to the following licenses/restrictions: The datasets generated and/or analyzed during the current study are not publicly available due to the following reasons: 1. Patient Privacy and Ethical Restrictions: The data contain sensitive personal health information (including medical images and pathological diagnoses) of patients. Public dissemination would compromise patient privacy and violate the ethical approval granted by the Ethics Committee of The Second Affiliated Hospital of Soochow University. 2. Institutional Data Governance Policy: The data are the property of The Second Affiliated Hospital of Soochow University] and are governed by its data protection policies. However, de-identified data may be made available to qualified researchers upon reasonable request. Data sharing requests must be submitted to the corresponding author (BZ, zhangbo_1122@126.com) and will be subject to review and approval by the aforementioned Ethics Committee to ensure that any data transfer complies with ethical and legal standards. Requests to access these datasets should be directed to BZ, zhangbo_1122@126.com.
