# Supplementary material for: Multiparametric-MRI habitat radiomics analysis for discriminating pathological types of brain metastases
Source: Front Oncol. 2025 Dec 2;15:1714315. doi: 10.3389/fonc.2025.1714315 (PMC12705402; doi:10.3389/fonc.2025.1714315)
Supplement: Supplementary file 1 [file Table1.docx]

Table 1.MR scanner details and imaging parameters.

| Parameters | Philips Achieva | Siemens Prisma | Philips Ingenia | Philips Achieva |
| --- | --- | --- | --- | --- |
|  | 1.5T | 3.0T | 3.0T | 3.0T |
| **CE-T1WI** |  |  |  |  |
| TR (ms) | 487.62 | 250 | 260 | 220 |
| TE (ms) | 15 | 2.49 | 4.61 | 1.76 |
| Matrix | 256×256 | 320×320 | 512×512 | 256×256 |
| FOV(mm^2^) | 230×230 | 220×220 | 230×230 | 230×230 |
| Layer thickness(mm) | 6 | 4mm | 5mm | 6mm |
| Slice gap(mm) | 7 | 5.2mm | 6.5mm | 7mm |
| pixels spacing | 0.90×0.90 | 0.69×0.69 | 0.45×0.45 | 0.45×0.45 |
| **FLAIR** |  |  |  |  |
| TR (ms) | 6000 | 9000 | 7000 | 6800 |
| TE (ms) | 120 | 81 | 120 | 131.73 |
| Matrix | 256×256 | 320×270 | 384×384 | 528×528 |
| FOV(mm^2^) | 230×230 | 230×230 | 230×230 | 232×232 |
| Layer thickness(mm) | 6 | 4mm | 5mm | 6mm |
| Slice gap(mm) | 7 | 4.8mm | 6.5mm | 7mm |
| pixels spacing | 0.90×0.90 | 0.72×0.72 | 0.60×0.60 | 0.44×0.44 |
| **T1WI** |  |  |  |  |
| TR (ms) | 487.62 | 250 | 250 | 241.51 |
| TE (ms) | 15 | 2.49 | 2.3 | 4.61 |
| Matrix | 256×256 | 320×270 | 512×512 | 256×256 |
| FOV(mm^2^) | 230×230 | 230×230 | 230×230 | 230×230 |
| Layer thickness(mm) | 6mm | 4mm | 5mm | 6mm |
| Slice gap(mm) | 7mm | 5.2mm | 6.5mm | 7mm |
| pixels spacing | 0.90×0.90 | 0.72×0.72 | 0.45×0.45 | 0.45×0.45 |
| **T2WI** |  |  |  |  |
| TR (ms) | 6000 | 4500 | 2800 | 2300 |
| TE (ms) | 120 | 98 | 91.22 | 90 |
| Matrix | 256×256 | 640×540 | 480×480 | 704×704 |
| FOV(mm^2^) | 230×230 | 230×230 | 230×230 | 232×232 |
| Layer thickness(mm) | 6 | 4mm | 5mm | 6mm |
| Slice gap(mm) | 7 | 5.2mm | 6.5mm | 7mm |
| pixels spacing | 0.90×0.90 | 0.36×0.36 | 0.48×0.48 | 0.33×0.33 |
